# Health-related quality of life in adults with von Willebrand disease: results of the French real-life Willebrand study on health-related quality of life

**DOI:** 10.1016/j.rpth.2025.103324

**Published:** 2025-12-30

**Authors:** Annie Borel-Derlon, Agnès Veyradier, Yohann Repessé, Nathalie Itzhar-Baïkan, Dominique Desprez, Fabienne Genre-Volot, Hervé Chambost, Laurent Ardillon, Brigitte Pan-Petesch, Sophie Bayart, Malika Barthez-Toullec, Grégory Marin, Marie Hélène André-Bonnet, Sophie Susen, Sylvia von Mackensen, Jenny Goudemand

**Affiliations:** 1 Hematology and Hemostasis Department, French Reference centre for von Willebrand disease, Haemophilia Treatment Centre, Caen, France; 2 Hematology Department, French Reference centre for von Willebrand disease, Haematology Unit, Lariboisière Hospital, Paris, France; 3 Hematology Department, Haemophilia Treatment Centre, Strasbourg, France; 4 Hematology Department, Haemophilia Treatment Centre, Dijon, France; 5 Pediatric Hematology and Oncology Department, Haemophilia Treatment Centre, Marseille, France; 6 Hematology Department, Haemophilia Treatment Centre, Tours, France; 7 Hematology Department, Haemophilia Treatment Centre, Brest, France; 8 Pediatric Hematology Department, Haemophilia Treatment Centre, Rennes, France; 9 Department of Clinical Development and Medical Affairs, Laboratoire français du Fractionnement et des Biotechnologies (LFB), Les Ulis, France; 10 Department of Statistics, Aixial, Boulogne-Billancourt, France; 11 Hematology and Transfusion Department, French Reference centre for von Willebrand disease, Haemophilia Treatment Centre, Lille, France; 12 Department of Medical Psychology, University Medical Centre, Hamburg-Eppendorf, Germany

**Keywords:** adult, observational study, quality of life, von Willebrand Disease, women

## Abstract

**Background:**

Hemorrhagic events in von Willebrand disease (VWD) impair patients’ physical health, daily functioning, and psychological/emotional well-being. While few studies have assessed health-related quality of life (HRQoL) in VWD, no prospective evaluation had been conducted in France.

**Objectives:**

The Willebrand study on HRQoL (WiSH-QoL) is an observational and prospective study that addressed this gap. Conducted in 27 French VWD treatment centers, it employed both generic and VWD-specific patient-reported outcome measures (PROs).

**Methods:**

Eligible patients included all ages and VWD types (type 1 restricted to basal von Willebrand factor antigen < 30 IU/dL). PROs (SF-36, VWD-QoL, and VWD-SAT) were assessed at baseline and 24 months.

**Results:**

In total, 224 adult patients were enrolled. Compared with the French general population, participants showed significantly reduced mental/emotional health and social/physical functioning. The VWD-specific PROs confirmed substantial physical impact in severe disease, including limitations in sports, leisure, and work. They also identified social impacts related to self-perception and relationships (family, others, and professionals). Physical and emotional well-being was particularly affected in women. Regardless of VWD type, patients reported mental health impacts, notably concerning future outlook. Social health deteriorated over time.

**Conclusion:**

The Willebrand study on HRQoL, using disease-specific PROs, reveals the real-life physical, emotional, and social burden of VWD, notably in severe forms and among women. By selecting key questions from these tools, clinicians can better assess these impacts across all patients and provide more comprehensive, long-term support for their well-being.

## Essentials

• HRQoL evaluation in French VWD adults is lacking.
• The prospective Willebrand study on HRQoL (WiSH-QoL) is using generic and disease-specific patient-reported outcomes.
• VWD reduces physical and mental HRQoL, with an increasing burden for severe types and in women.
• VWD impacts adults’ daily life, including work, sport, and leisure and view of future life.

## 1 Introduction

Recurrent hemorrhagic episodes in hereditary bleeding disorders, particularly von Willebrand disease (VWD), are recognized to not only cause significant physical consequences but also potentially leading to psychological, emotional, and social sequelae [1,2]. To evaluate the comprehensive impact of these bleeding disorders on patients’ daily functioning, several specialized instruments have been developed, including health-related quality of life (HRQoL) questionnaires.

Most prior studies on HRQoL in adults with VWD relied solely on generic questionnaires [[3], [4], [5]], with a predominant focus on women [[6], [7], [8], [9], [10]] or patients with severe joint damage requiring replacement surgery [11,12]. The Willebrand study on HRQoL (WiSH-QoL) was designed as a comprehensive, state-of-the-art investigation of HRQoL in patients with VWD living in France. It combined a generic HRQoL questionnaire with a newly developed VWD-specific tool, evaluating physical, mental, and social dimensions. The generic questionnaire enabled comparisons with the French general population, while the disease-specific instrument provided an in-depth assessment of the challenges associated with living with VWD. Participants were enrolled irrespective of age, gender, or disease severity and followed prospectively for 2 years to identify domains of QoL that might be more affected by bleeding events or disease evolution during the study.

By exploring not only the physical but also the emotional and social effects of the disease on their daily lives over a 2-year-follow-up period, this study aimed to provide in real-life valuable and accurate information for understanding patient health or HRQoL evolution disparities due to VWD types or gender. Ultimately, this could enhance the VWD journey for both patients and clinicians in France.

## 2 Material and Methods

### 2.1 Study design and population

WiSH-QoL is a prospective and observational study conducted in France (EUPAS107760) in accordance with international standards and national data protection regulations. The study was proposed to all patients with any VWD type (type 1 restricted to von Willebrand factor antigen [VWF:Ag] < 30 IU/dL), of any age or gender, and enrolled during their routine outpatient’s visits in their treatment centers. Participants were followed up for 24 months (M24: −3 or +6 months allowed). No visit schedule was predefined, visits at 6, 12, and 18 months could be collected if performed. Diagnosis of VWD and type classification were at the discretion of the investigators. All treatment decisions were made by the treating physicians in accordance with current guidelines and were not influenced by the study protocol. All patients provided written informed consent prior to their enrolment.

Within this study, only data from adult patients (≥18 years) were analyzed. Pediatric data were reported separately [13]. In order to be representative of the target population, it was planned to recruit 25% of the adult cohort of the national French VWD registry, corresponding to 240 patients at the start of the study in 2014.

### 2.2 Data collection

Sociodemographic data (eg, age, gender, body mass index, viral status, concomitant treatments, and associated pathologies) and VWD status (a VWD history with VWD diagnosis, a history of joint lesions or gastrointestinal bleeds, and therapeutic strategies) were collected from patients’ medical records and entered into an electronic case report. Bleeding severity was collected from the medical chart at inclusion (M0) and M24 if available, using the total score of the Tosetto bleeding score (BS), the score most commonly used by clinicians in France at the start of the study [14]. Additionally, the 2 items of the bleeding assessment tool of the International Society on Thrombosis and Haemostasis/Scientific and Standardization Committee (ISTH-BAT/SCC) related to menorrhagia and postpartum hemorrhage were collected for women [15].

During the follow-up, all medical events were collected including minor or major (treated in hospital for >3 days) bleeds, surgeries (including deliveries), and invasive procedures. The administration of VWF, on-demand or long-term prophylaxis (LTP), as well as the other drugs commonly used to treat patients with VWD (eg, desmopressin [DDAVP], tranexamic acid, iron, and estrogen) were recorded when available. Patient-reported outcome (PRO) questionnaires were completed at M0 and M24, in paper format, during the patient visits or at home (depending on the site organization) and then entered into the database by a data-entry operator.

#### 2.2.1 PRO measures

PRO questionnaires assessed the HRQoL, using self-administered generic (SF-36V2) and VWD-specific (VWD-QoL) questionnaires, and a global treatment satisfaction (VWD-SAT), regardless of treatment administered and modalities. SF-36 consists of 36 items divided into 8 dimensions (underlying concepts in Supplementary Table 1) [16,17] and summarized by 2 scores: physical component summary (PCS) and mental component summary (MCS) [18]. Each scale is transformed into a 0 to 100 scale with higher scores indicating better HRQoL.

VWD-QoL and VWD-SAT were originally developed in Italy [19]. The French version was linguistically validated by the MAPI institute (Lyon, France), including forward/backward translation, developer’s and clinicians review followed by cognitive interviews on a sample of 5 adult patients. The original VWD-QoL consists of 85 items, rated from 1 (never) to 5 (always), divided into 13 dimensions (underlying concepts in Supplementary Table 2). All dimensions equally contribute to a total score. Items are transformed to a 0 to 100 scale. The VWD-SAT, a disease-specific treatment satisfaction questionnaire consists of 34 items, rated from 1 (totally agree) to 5 (totally disagree), and then transformed to a 0 to 100 scale, divided into 6 dimensions and summarized by a total score. The higher scores, the lower HRQoL or lower treatment satisfaction.

Additional questions were separately asked regarding patients’ sociodemographic status, place of residence, educational level (measured by the International Standard Classification of Education) [20], potential disabilities, absenteeism, and exploratorily analyzed as covariates.

### 2.3 Statistical methods

Qualitative variables were described in terms of numbers and percentages. These were compared using chi-squared tests or Fisher exact tests if at least one of the theoretical values was <5. Missing values were not considered in the calculation of percentages, and their number was indicated.

Quantitative variables were described either by their mean/SD for normally distributed continuous variables or by their median/range for nonnormally distributed variables. These were compared using Student’s *t*-tests if the variable’s distribution was Gaussian, and either Wilcoxon–Mann *U*–Whitney (when comparing 2 groups) or Kruskal–Wallis tests (when comparing >2 groups) if not Gaussian. Normality of the distribution was assessed using Shapiro–Wilks tests.

Specific missing data rules were adopted for each PRO questionnaire (Supplementary Table 3). All analyses were carried out for the entire cohort and compared across VWD types (1, 2 [pooled whatever the subtypes], 3, or unknown [ie, not yet classified within a specific VWD type]). Subgroup analyses were carried out according to gender for 18- to 50-year-old participants, treatment regimen (LTP or on-demand) for patients with type 3 VWD, and severity of VWF deficiency (VWF:RCo ≥ 15 or < 15 IU/dL) for types 1, 2, and unknown. PRO questionnaire total scores were compared between patients treated or not with iron therapy and between women (18 to 50 year olds) treated or not with hormone therapy.

The SF-36 scores were compared with the average data for the general French population after weighting the literature values [21] by age and gender group to obtain SF-36 dimension scores comparable with the scores from the WiSH-QoL study. The correlation between ISTH-BAT-menorrhagia and PRO questionnaire total scores for women was evaluated using the Spearman coefficient.

Three exploratory multivariate linear regression models were carried out to test the sensitivity of both generic and specific questionnaires, analyzing the relationship between the 2 SF-36 components scores (PCS and MCS) or the total VWD-QoL score and selected sociodemographic and clinical covariates (description in Supplementary Table 4). All statistical analyses were performed using SAS Entreprise Guide V7.12 (SAS Institute).

## 3 Results

### 3.1 Study population

**Figure 1:**
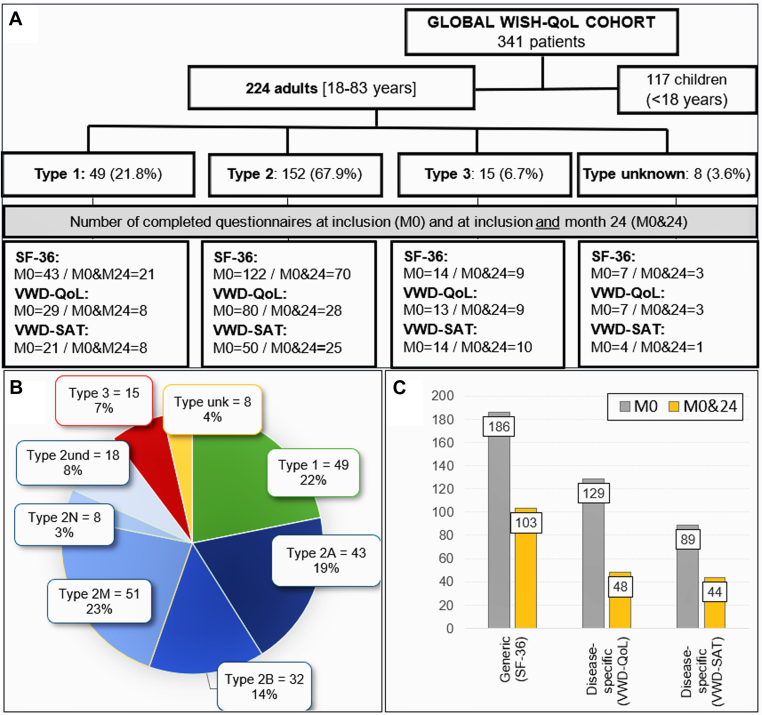
Adult flow chart by VWD type and completed PRO questionnaires. (A) Adult flow diagram according to VWD types. (B) Description of VWD types and subtypes in adult patients with VWD (*n* = 224). (C) Number of PRO questionnaires completed at M0 and at M0 and 24. M0, participants who completed the questionnaires at inclusion; M0&24, participants who completed both inclusion (M0) and end-of-study (M24) questionnaires; HRQoL, health-related quality of life; PRO, patient-reported outcome; SF-36, chronic-generic HRQoL questionnaire; und, undetermined; unk, unknown; VWD-QoL, disease-specific HRQoL questionnaire for patients with VWD; VWD-SAT, disease-specific treatment satisfaction questionnaire for patients with VWD; VWD, von Willebrand disease.

From October 2014 to November 2017, 341 eligible patients were enrolled in the global WiSH-QoL cohort by 27 French VWD treatment centers with the last follow-up visit performed in January 2020. Among them, 224 patients (65.7%) were adult patients (Figure 1A). They mainly presented with a type 2 VWD (67.9%), followed type 1 (21.8%), type 3 (6.7%), and unknown type (3.6%). Type 2 subtypes are shown in Figure 1B. Overall, approximately 50% of patients displayed a severe VWD phenotype with a VWF:RCo level < 15 IU/dL (Table 1).

**Table 1:** Coagulation factors and platelet count at inclusion (M0).

Parameters at M0 (not at disease onset)^a^	Type 1 (*n* = 49)	Type 2 (*n* = 152)	Type 3 (*n* = 15)	Type unknown (*n* = 8)	All patients (*N* = 224)
Factor VIII (IU/dL)	33.5 (5-100)	42.5 (2-202)^b^	3.0 (1-57)	43.5 (12-97)	40.0 (1-202)
Missing (*n*)	1	0	0	0	1
VWF:Ag (IU/dL)	20 (6-46)	33 (6-144)	2 (1-24)	28 (8-46)	27 (1-144)
Missing or below limit of detection (*n*)	0	1	10	0	11
VWF:RCo (IU/dL)	21 (1-40)	16 (5-124)	5 (0-9)	25 (9-31)	17 (0-124)
Missing or below limit of detection (*n*)	6	25	11	2	44
VWF:RCo < 15 IU/dL	18 (36.7)	75 (49.3)	15 (100.0)	3 (37.5)	111 (49.6)
Platelets <150 G/L	NR	Type 2B (*n* = 32); 7 (22)	NR	NR	NR

Values are *n* (%) or median (minimum-maximum).

Ag, Antigen; NR, not relevant for other von Willebrand disease types than type 2B; VWF, von Willebrand factor; RCo, ristocetin cofactor.

a The rate measured at inclusion is not necessarily the baseline rate (recent replacement therapy).

b Included 8 patients with type 2N VWD, among whom 4 presented with factor VIII of < 20 IU/dL.

The patient’s characteristics at inclusion are displayed according to VWD type in Table 2. Their median age at inclusion was 42 years (range, 18-83 years) and the median time since VWD diagnosis was 18 years (range, 0.04-60 years). Most of them were females (71%), and among these women, 116 (73%) were between the ages of 18 and 50 years.

**Table 2:** Patient characteristics at inclusion (M0) according to VWD type.

Demographic and clinical characteristics at inclusion	Type 1, *n* = 49 (21.8%)	Type 2, *n* = 152 (67.9%)	Type 3, *n* = 15 (6.7%)	Type unk^a^, *n* = 8 (3.6%)	All, *N* = 224 (100%)
Gender					
Male	9	50	4	2	65
Female	40	102	11	6	159
Ratio	0.2	0.5	0.4	0.3	0.4
Age (y)					
At VWD diagnosis	21 (0.5-60)	22 (0-78)^b^	1 (0-17)^b^	26 (1.0-60)	20 (0-78)^b^
At first substitutive treatment	26.5 (0.5-74)	30 (1-78)	4.8 (0-41)	20.5 (4-63)	27 (0-78)
At inclusion in WiSH-QoL	40 (18-78)	43 (18-83)	35 (18-69)	42 (19-70)	42 (18-83)
Time since diagnosis (y)					
Median (minimum-maximum)	16 (0.5-58)	19 (0.04-60)	32 (18-53)	11 (0.2-30)	18 (0.04-60)
Diagnosis circumstances					
VWD familial history	24 (49)	64 (42)	2 (13)	3 (38)	93 (41)
Bleeding event	13 (27)	54 (36)	12 (80)	1 (13)	80 (36)
Blood test anomaly	12 (25)	27 (18)	0	4 (50)	43 (19)
Unknown	0	7 (5)	1 (7)	0	8 (4)
Tosetto bleeding score^c^					
Abnormal value	34 (69)	122 (82)	14 (93)	5 (63)	175 (79)
Median (minimum-maximum)	6 (0-19)	8 (0-24)^d^	26 (3-31)	4 (1-20)	8 (0-31)^d^
Men	6 (0-12); 9	7 (0-21); 48	27 (11-31); 4	3 (3-3); 2	7 (0-31); 63
All women	7 (1-19); 40)	8 (0-24); 101)	26 (3-29); 11)	5 (1-20); 6)	8 (0-29); 158*)*
Women, 18-50 y olds	6 (1-16); 28	8 (0-22); 72	26 (12-29); 9	5 (1-20); 6	8 (0-29); 115
Women, 18-50 y olds: ISTH scores					
ISTH-menorrhagia^e^	2 (0-4); 27)	2 (0-4); 68)	4 (2-4); 8)	1 (0-2); 4)	2 (0-4); 107)
ISTH-PPH^f^	0 (0-3); 18)	2 (0-3); 40)	2 (0-3); 4)	0 (0-0); 2)	1 (0-3); 64)
At least 1 symptom					
Joint lesion	1 (2)	2 (1)	12 (80)	0	15 (7)
Gastrointestinal hemorrhage	5 (10)	20 (13)	8 (53)	1 (13)	34 (15)
Hemoglobin (g/dL): abnormal value	1 (2)	9 (6)	0	0	10 (5)
Median (minimum-maximum)	14 (9-16)	14 (9-17)	14 (12-16)	14 (12-16)	14 (9-17)
Medication					
Iron therapy	10 (20)	42 (28)	5 (33)	3 (38)	60 (27)
Men (*n* = 65)	0 (0)	11 (22)	0 (0)	0 (0)	11 (17)
Women (*n* = 159)	10 (25)	31 (30)	5 (45)	3 (50)	49 (31)
Estrogen and/or progestogen therapy					
All women (*n* = 159)	14 (35)	49 (48)	8 (73)	3 (50)	74 (47)
Women 18-50 y olds (*n* = 115)	12 (43)	34 (47)	6 (67)	3 (50)	55 (48)
Antifibrinolytics	13 (27)	60 (39)	10 (67)	3 (63)	86 (38)
Local hemostatics	1 (2)	1 (0.7)	0 (0)	1 (13)	3 (1)
DDAVP-nasal	2 (4)	22 (14)	0 (0)	0 (0)	24 (11)
DDAVP- IV	3 (6)	4 (3)	0 (0)	1 (13)	8 (4)
Long-term VWF prophylaxis	2 (4)	2 (1)	9 (60)	0 (0)	13 (6)

Values are *n* (%) or median (minimum-maximum).

DDAVP, desmopressin; IV, intravenous; ISTH, International Society on Thrombosis and Haemostasis/Scientific and Standardization Committee; PPH, postpartum hemorrhage.

a Type unk (type unknown) was defined as patients not classified within a specific VWD type.

b 0 = neonatal diagnosis.

c Tosetto bleeding score: ranging from −3 (no spontaneous bleeding symptom, no bleeding after tooth extraction, surgery, or delivery) to +45 (major bleeding for all symptoms).

d 3 missing data.

e ISTH-menorrhagia score: grading from 0 (no/trivial) to 4 (acute menorrhagia requiring hospital admission and emergency treatment or requiring blood transfusion, replacement therapy, desmopressin or requiring dilation and curettage or endometrial ablation or hysterectomy).

f ISTH-PPH score: grading from 0 (no/trivial) to 4 (any procedure requiring critical care or surgical intervention [eg, hysterectomy, internal iliac artery legation, uterine artery embolization, and uterine brace sutures]), only asked to women who have given birth.

The Tosetto BS was abnormal in 79% of patients and is displayed in Table 2 according to VWD type and gender. As expected, the highest score was observed in patients with type 3 VWD. No significant difference in Tosetto score was observed between the overall female cohort (*n* = 158) and the subgroup of women aged 18 to 50 years (*n* = 115), a finding consistent across all types of VWD. ISTH-BAT-menorrhagia and postpartum hemorrhage scores were also the highest in women of childbearing age with type 3 VWD. For the 159 patients for whom data were available at both M0 and M24, the median Tosetto BS was constant over time (M0: 8 [range, 0-31], M24: 9 [range, 0-33]), regardless of gender (not shown).

Only 10 patients had abnormal hemoglobin levels but none with a clinical significance. This may reflect frequent iron supplementation (60 patients—27% of the cohort); among them, 49 were women (82%), and 5 had type 3 VWD (8%). Moreover, 47% of women were undergoing hormone treatment (Table 2).

The main treatments administered during the 2 years of follow-up were tranexamic acid (38%), nasal DDAVP (11%), and hormone therapy (47% of women). Thirteen patients (6%), including 9 with type 3 VWD, were on LTP, mainly due to the recurrence of joint damage or hemarthrosis (62%), gastrointestinal hemorrhage or angiodysplasia (23%), and epistaxis and/or metro/menorrhagia (15%).

More than two-third of patients (69%) had at least 1 comorbidity, with older patients being more affected than younger ones (Supplementary Table 5) and without significant difference between the VWD types (not shown). Their sociodemographic characteristics are displayed in Supplementary Table 6. The mean follow-up time in the study was 20.6 ±10.1 months [up to 39.5 months]. Finally, 74% of participants had a completed 2-year-follow-up and 37% of patients with type 1 VWD, 23% with type 2, 7% with type 3, and 50% with type unknown did not return at 24 months.

Clinical events occurred in 60% of patients during follow-up, more in patients with type 3 VWD (100%) than that in those with type 2 (63%) or type 1 (43%) VWD (Supplementary Table 7). Most of them were minor bleeds (49%), followed by surgeries (24%) and invasive procedures (21%). Very few events (6%) were major bleeds, mainly gastrointestinal (72%) or perinatal (8%). DDAVP was administered for 6% of treated minor bleeding. Substitutive VWF concentrate was administered for 72% of treated minor bleeding, 92% of major bleeding, 81% of surgeries, and 64% of invasive procedures. Ninety-four (42%) patients received at least 1 injection of VWF concentrate. No adverse events related to substitutive therapy occurred in patients throughout the study.

### 3.2 PRO results

The number of PRO questionnaires completed during the study is displayed for all adults by VWD types and by time in Figure 1A, C. Not all participants completed an HRQoL questionnaire at M0 and even less at M24, with fewer specific VWD-QoL than SF-36 questionnaires (58% vs 83% at M0 and 38% vs 68% at M24, respectively). Whatever the visit, the nonresponders were more patients with type 1 or 2 VWD than those with type 3.

### 3.3 HRQoL evaluation with the SF-36 generic questionnaire

**Figure 2:**
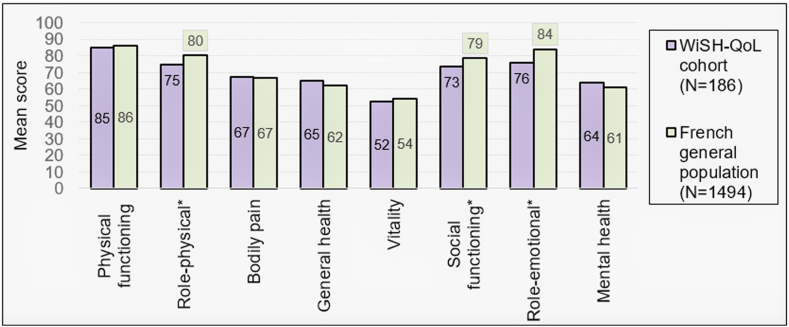
SF-36: Willebrand study on health-related quality of life (WiSH-QoL) cohort vs French general adult population. SF-36: the higher the score, the better the health-related quality of life. ∗Statistically significant: role-physical, *P* = .007; social functioning, *P* = .004; role-emotional, *P* < .001.

The comparison of the SF-36 results with those of the general French population showed significantly lower scores (*P* < .05) for patients with VWD in 3 of the 8 dimensions (role-physical, role-emotional, and social functioning), highlighting the negative impact of VWD on patients’ physical and social activities as well as mental well-being (Figure 2).

Analysis of VWD types revealed that the 4 PCS scores were the most discriminative, being lowest in type 3 and highest in type 2 disease (Table 3). No significant intertype differences were found for the 4 MCS components. Furthermore, when categorizing patients with type 1, type 2 (excluding 2N), and unknown VWD by VWF:RCo levels, only the role-physical score was significantly superior in the cohort with VWF:RCo of ≥ 15 IU/dL. No significant gender-based differences in SF-36 scores were detected in the 18- to 50-year age group, although a nonsignificant trend indicated that women scored lower the vitality dimension, as well as in the aggregate MCS.

**Table 3:** VWD-QoL results at M0 according to VWD type, VWF:RCo levels, gender of patients aged 18 to 50 y or over time (M0 vs M24).

SF-36	VWD type (*n* = 186)						VWF:RCo (type 3 and 2N excluded) (*n* = 167)				Gender (18-50 y olds) (*n* = 129)			M0 vs M24 (*n* = 103)		
Mean score	All (*n* = 186)	Type 1 (*n* = 44)	Type 2 (*n* = 122)	Type 3 (*n* = 13)	Type unk (*n* = 7)	*P*	All (*n* = 167)	<15 IU/dL (*n* = 77)	≥15 IU/dL (*n* = 90)	*P*	Men (*n* = 33)	Women (*n* = 96)	*P*	M0 (*n* = 103)	M24 (*n* = 103)	*P*
Physical functioning	85	87	88	54	79	**<.001**	87	84	90	NS	87	87	NS	83	84	NS
Role-physical	75	76	77	62	53	**.015**	75	70	80	**.028**	77	77	NS	74	75	NS
Bodily pain	67	64	72	49	51	**.015**	69	67	71	NS	73	69	NS	66	65	NS
General health	65	65	68	46	58	**.011**	66	66	67	NS	63	66	NS	64	65	NS
Vitality	52	53	53	46	54	NS	53	53	53	NS	58	50	NS	52	51	NS
Social functioning	73	72	75	63	66	NS	74	74	74	NS	73	74	NS	74	73	NS
Role-emotional	76	77	78	64	52	NS	77	73	80	NS	80	77	NS	74	74	NS
Mental health	64	65	64	58	64	NS	64	64	64	NS	68	63	NS	63	64	NS
PCS	51	51	53	42	47	**.004**	52	51	53	NS	52	52	NS	51	51	NS
MCS	44	45	45	43	42	NS	44	44	45	NS	46	44	NS	44	44	NS

SF-36: the higher the score, the better the health-related quality of life. Bold values indicate statistical significance.

M0, inclusion visit; M24, end-of-study visit (range, 21-30 mo); MCS, mental component score (summary score of vitality, social functioning, role-emotional, and mental health); NS, not statistically significant; PCS, physical component score (summary score of physical functioning, role-physical, bodily pain, and general health); RCo, ristocetin cofactor; unk, unknown; VWD, von Willebrand disease; VWF, von Willebrand factor.

In a focused analysis of patients with type 3 VWD, those receiving LTP (*n* = 7) demonstrated a significantly poorer PCS than those managed on-demand (*n* = 6; 33 vs 51; *P* = .0184). No differences in mental health scores were apparent. Finally, longitudinal assessment of the 103 patients who completed surveys at both baseline and 24 months showed no statistically significant temporal changes in SF-36 outcomes (Table 3).

### 3.4 HRQoL evaluation with the VWD-specific questionnaire

Using the VWD-QoL reveals more differences between categories. Among all patients assessed with the VWD-QoL, the most impacted dimension was future—a mental health-related domain where higher scores indicate lower HRQoL (Table 4). The mean total score showed greater impairment of HRQoL in patients with type 3 than that in those with the other types, as they were higher affected on 8 dimensions, including 2 related to their physical health (physical and sport), 3 related to their mental health (feeling, view, and future), and 3 related to their social health (family, others, and work). Patients with type 1 or type 2 VWD had similar HRQoL; however, when they were compared according their VWF:RCo levels, those with <15 IU/dL reported more difficulties with leisure and sport activities. Interestingly, despite the small sample size (*n* = 9), patients with type 1 appeared more affected by therapeutic constraints than those with type 3, even though the latter receive more intensive treatment.

**Table 4:** VWD-QoL results at M0 according to VWD type, VWF:RCo levels, gender of patients aged 18 to 50 y, or over time (M0 vs M24).

VWD-QoL	VWD type (n = 129)						VWF: RCo (type 3 and 2N excluded) (*n* = 113)				Gender (18-50 y olds) (*n* = 103)			M0 vs M24 (*n* = 48)		
Mean score	All (*n* = 129)	Type 1 (*n* = 30)	Type 2 (*n* = 80)	Type 3 (*n* = 12)	Type unk (*n* = 7)	*P*	All (*n* = 113)	<15 IU/dL (*n* = 54)	≥15 IU/dL (*n* = 59)	*p-value*	Men (*n* = 30)	Women (*n* = 73)	*P*	M0 (*n* = 48)	M24 (*n* = 48)	*P*
Treat	25 (*n* = 60)	36 (*n* = 9)	20 (*n* = 37)	33 (*n* = 12)	36 (*n* = 2)	**.010**	24 (*n* = 46)	26 (*n* = 25)	20 (*n* = 21)	NS	21 (*n* = 16)	28 (*n* = 34)	NS	25 (*n* = 26)	25 (*n* = 28)	NS
Complaint	13 (*n* = 60)	23 (*n* = 9)	12 (*n* = 37)	9 (*n* = 12)	19 (*n* = 2)	NS	15 (*n* = 46)	14 (*n* = 25)	16 (*n* = 21)	NS	8 (*n* = 16)	15 (*n* = 34)	NS	13 (*n* = 26)	12 (*n* = 28)	NS
Physical	24	23	20	52	28	**.012**	21	24	19	NS	17	27	**.005**	27	27	NS
Feeling	23	23	21	39	21	**.032**	21	24	19	NS	19	25	NS	24	23	NS
View	25	24	23	45	19	**.004**	23	23	23	NS	21	27	NS	27	27	NS
Family	21	17	18	50	24	**.002**	18	20	16	NS	21	22	NS	23	23	NS
Others	25	25	23	39	35	**.006**	24	27	24	NS	22	27	NS	23	27	**.019**
Sport	26	20	24	61	26	**<.001**	23	28	19	**.042**	29	26	NS	30	33	NS
Work	13	14	10	39	7	**<.001**	11	11	11	NS	12	15	NS	17	14	NS
Deal	28	31	27	37	22	NS	27	28	26	NS	27	29	NS	28	26	NS
Hospital	15	16	13	24	15	NS	14	15	14	NS	13	17	NS	13	12	NS
Future	33	31	29	65	28	**<.001**	30	31	28	NS	28	36	NS	35	35	NS
Sex	18	13	18	32	11	NS	17	19	15	NS	20	16	NS	19	26	**.027**
Total	22	23	20	38	21	**<.001**	21	22	19	NS	19	24	NS	23	23	NS

VWD-QoL: the higher the score, the lower the HRQoL. Bold values indicate statistical significance.

HRQoL, health-related quality of life; M0, inclusion visit; M24, end-of-study visit (range, 21-30 mo); NS, not statistically significant; RCo, ristocetin cofactor; unk, unknown; VWD, von Willebrand disease; VWF, von Willebrand factor; VWD-QoL, disease-specific HRQoL questionnaire for patients with VWD.

When comparing scores between men and women of the same age, men appeared less affected than women in most areas, particularly in the physical domain. Women’s scores were only slightly better than men’s in the sports dimension. Over time, the scores showed a significant deterioration in HRQoL in 2 dimensions related to social health (others and sex) at M24 (Table 4).

### 3.5 VWD-specific treatment satisfaction

Patients reported the highest treatment satisfaction in centers and specialist dimensions, but the lowest in burden and efficacy. While no significant differences emerged across VWD types overall, patients with type 2 VWD expressed slightly higher satisfaction across all dimensions—particularly for ease of treatment use (Table 5). Only burden and general dimensions showed marginally better scores for men versus women. Scores remained stable over time except for efficacy, which demonstrated significant improvement by month 24.

**Table 5:** VWD-SAT results at M0 according to VWD type, VWF:RCo levels, gender of patients aged 18 to 50 y, or over time (M0 vs M24).

VWD-SAT	VWD type (*n* = 89)						VWF: RCo (type 3 and 2N excluded) (*n* = 74)				Gender (18-50 y olds) (*n* = 66)			M0 vs M24 (*n* = 44)		
Mean score	All (*n* = 89)	Type 1 (*n* = 22)	Type 2 (*n* = 50)	Type 3 (*n* = 13)	Type unk (*n* = 4)	*P*	All (*n* = 74)	<15 IU/dL (*n* = 41)	≥15 IU/dL (*n* = 33)	*P*	Men (*n* = 17)	Women (*n* = 49)	*P*	M0 (*n* = 44)	M24 (*n* = 44)	*P*
Ease	23	27	18	31	29	**.014**	22	21	24	NS	20	22	NS	23	24	NS
Efficacy	26	29	23	25	31	NS	26	25	27	NS	24	26	NS	27	23	**.012**
Burden	26	29	23	37	28	NS	25	24	26	NS	18	27	NS	27	25	NS
Specialist	10	10	9	11	16	NS	10	10	9	NS	10	9	NS	11	12	NS
Centre	11	11	9	17	16	NS	10	10	10	NS	12	10	NS	12	11	NS
General	17	19	15	18	25	NS	17	16	18	NS	13	18	NS	16	17	NS
Total	19	21	16	25	24	NS	18	18	19	NS	17	19	NS	20	19	NS

VWD-SAT: the higher the score, the lower the treatment satisfaction. Bold values indicate statistical significance.

M0, inclusion visit; M24, end-of-study visit (range, 21-30 mo); NS, not statistically significant; RCo: ristocetin cofactor; unk, unknown; VWD-SAT, treatment satisfaction for patients with VWD; VWD, von Willebrand disease; VWF, von Willebrand factor.

### 3.6 Potential confounding factors

Although iron deficiency’s potential impact on PROs was not analyzed due to the low prevalence of abnormal hemoglobin levels (4.5% of the cohort), patients receiving iron supplementation (*n* = 53) showed a trend toward lower SF-36 mental scores (mean MCS, 42) than untreated patients (*n* = 133; mean MCS, 45; *P* = .07). Notably, iron treatment was primarily administered to women (82%). Other PROs (PCS, VWD-QoL, and VWD-SAT) showed no significant differences between treated and untreated patients.

Moreover, for the 18- to 50-year-old women, we observed that menorrhagia had no potential impact on their PRO questionnaire results as no correlation was found between the ISTH-BAT-menorrhagia score and PRO questionnaire total scores: intraclass correlation coefficient, −0.00098, −0.06695, and 0.10239 for PCS (*n* = 88), MCS (*n* = 89), and VWD-QoL (*n* = 68) respectively, with coefficient of 1 being the best correlation. In addition, the comparison of PRO questionnaire total scores did not reveal any significant differences between the responses of women treated or not with hormone therapy.

The exploratory multivariate analysis performed on both SF-36 component scores (PCS and MCS) showed that having been diagnosed a long time ago, having at least 1 joint lesion, and not having an active professional status (including being off work for a long period) were significantly associated with a greater impact on PCS. No variables were shown to significantly influence the MCS. With the total VWD-QoL score**,** the exploratory analysis showed that VWD type, especially type 3, being a woman, and having a disability status, were significantly associated with a greater impact on the HRQoL (Supplementary Table 8).

## 4 Discussion

### 4.1 Patient population

This WiSH-QoL study is the first conducted in France focusing on HRQoL of patients living with VWD. It involved 341 patients, including 224 adults, recruited from 27 centers across the country. The objective was to include approximately one-quarter of the adult patient population identified in the French VWD registry at the study’s outset (240 patients), a target that was nearly met, thereby supporting the representativeness of the WiSH-QoL cohort. The cohort comprises a large majority of women (71%), which is consistent with previous QoL studies in VWD: 62% in a Dutch cohort [4], 73% in a Danish study [22], 78% in a Canadian study [5], and even 83% in a German cohort [23]. In contrast, the distribution of types is markedly different from that typically reported. Patients in the WiSH-QoL cohort are predominantly those with type 2 VWD (68%), whereas several other studies [4,5,[22], [23], [24]] report a majority of patients with type 1 VWD (representing between 53% and 80% of cases). This divergence stemmed probably from our stringent inclusion criteria for type 1 VWD (VWF:Ag < 30 IU/dL), which was also the criterion used in the French VWD registry in 2014. The same distribution was observed in the pediatric cohort [13]. Furthermore, half of the patients included in the WiSH-QoL study have VWF:RCo levels < 15 IU/dL, corresponding to severe forms, whereas most patients included in several other studies [4,5,[22], [23], [24]] have mild to moderate forms. The retention rate of patients in the study (74%) was satisfactory with a mean follow-up time of 20.6 months as follow-up visits were not mandatory by nature of this noninterventional study. Notably, a high proportion of less symptomatic phenotype patients were among those who did not return.

### 4.2 VWD impacts physical, emotional, and social HRQoL compared with the French population

Given that the sociodemographic characteristics of this adult cohort are consistent with those of the French general population [21], the SF-36 results indicate that patients with VWD primarily experience physical limitations that impact their daily activities and work capacity. Interestingly, unlike the findings in Dutch, Canadian, and German cohorts [4,5,23], the impairment in the vitality dimension was not observed in French patients compared with that in the general population. Furthermore, their daily lives were characterized more by emotional and social limitations than by mental problems such as clinical anxiety or depression. It is noteworthy that this emotional impact was also absent in the Canadian study [5].

Another notable finding is that patients with VWD rated their mental and general health slightly higher than the French reference population. This may suggest that patients’ self-expectations and needs are subconsciously adapted to their chronic condition. Moreover, and in contrast again to the French population, no decline in mental health scores was reported over time, a stability potentially attributable to the shorter follow-up period of this study or to the consistency of patient responses over time [21].

### 4.3 Negative VWD impacts on physical health increase with the severity of the disease

Among the VWD types, patients with type 3 VWD constitute a distinct group: the diagnosis is established in childhood, they have very high bleeding scores, and they experience frequent joint involvement (80%) and gastrointestinal bleedings (53%), necessitating repeated replacement therapy. This clinical profile directly impacts their QoL, as reflected in the SF-36 questionnaire where they report the lowest physical capacity scores compared with patients with type 1 or 2 VWD, as well as in the physical and sport items of the VWD-QoL questionnaire. Surprisingly, these physical differences by VWD type were not seen with the SF-36 in the retrospective German WIL-QoL study, nor with the disease-specific questionnaire except for the sport dimension [23]. Furthermore, among patients without type 3 VWD, disease severity—defined by a VWF:RCo level of < 15 IU/dL—was associated with greater impairment. This was reflected in significantly poorer scores on the role-physical domain of the SF-36, indicating limitations in work and daily activities, and the sport domain of the VWD-QoL. These results resembled the experience of patients with severe bleeding disorders such as hemophilia [25] or afibrinogenemia [26]. Patients with type 2 generally reported better HRQoL than those with type 3 and an equal HRQoL to those with type 1.

Only patients with severe VWD were on LTP attesting the low rate of patients with VWD on LTP (6%), although it is slightly higher than the 1.6% reported in a European/US surveillance study [27]. The value of initiating LTP in patients with the most severe VWD has been advocated for many years, by analogy with the management of hemophilia as others have pointed out [[28], [29], [30]]. The subanalysis regarding patients with type 3 VWD on LTP vs on-demand showed poorer HRQoL on the SF-36 physical dimension probably reflecting the selection of more severely affected individuals. The same observation was made in the German cohort [23].

The gender-based analysis showed that SF-36 physical scores were similar in men and women, with only the general health dimension tending to be better in women at M0. However, only a 1-point difference was reported in the median Tosetto score between men and women, irrespective of VWD type, indicating that the men included probably had a high level of symptoms. These gender similarities were not seen in the general French population, where all raw scores for women were lower than those for men [21]. Nevertheless, both PCS and MCS were very similar to those previously described by de Wee et al. [4] in men and women with moderate and severe VWD. Contrary to previously published observations in women with heavy menstrual bleeding (HMB) [6,7,9], women in WiSH-QoL did not have significantly higher impairments in the bodily pain dimension.

### 4.4 VWD-specific questionnaires provides deeper insights on mental or social health

The disease-specific VWD-QoL questionnaire brought a unique perspective to the study. While the SF-36 assessment found no statistical differences in mental health between types, which was consistent with findings from other publications [[3], [4], [5],^23^], the VWD-QoL was able to reveal the mental impact of the disease, especially with regards to feeling about the future, regardless of the type of VWD. This impact was also seen in the Danish cohort [22], with the worse scores found in treatment, future, and view domains. Interestingly, compared with the Danish cohort, the French patients were more concerned about how to deal with the disease and their limitation to practice sport than about their perception of themselves and the relationship with their partner.

The social impact, also assessed in greater depth, was found to increase with the disease severity, affecting the self-perception of patients, relationships with family or others, and work. In contrast, this social impact was not seen in German study, for which the relationship, family, and other dimensions were the least affected by the disease, whatever the VWD type [23]. Moreover, the VWD-QoL scores of French patients with type 2, all subtypes pooled, most often indicated a similar or slightly better HRQoL than those of patients with type 1 (with VWF:Ag of < 30 IU/dL).

The use of VWD-specific PRO questionnaires also revealed the profound impact of VWD on women aged 18 to 50 years. While only the physical dimension was statistically lower in women at baseline, a consistent trend was observed where men scored better in almost all other dimensions (except for sports/leisure). In contrast, the German study found no such gender disparity, although its cohort had a greater female representation (83%) than the cohort assessed in this study (71%) [23].

Interestingly, the ISTH-menorrhagia score showed no correlation with the total VWD-QoL score, and the total score was similar between patients receiving or not receiving iron therapy. This finding contrasts with a recent study that used a 4-question assessment to demonstrate a significant impact of menstruations on daily activities (specifically school/work performance, physical and social activity, and intimacy) [31]. These specific domains were not captured by our questionnaires, which were designed to assess QoL in a broader, more comprehensive manner. It contrasts also with the findings of the WIL-QoL Study group [24] where women with self-reported often/all the time HMB reported worse overall VWD-QoL score compared with those with never/rarely HMB [24].

Although the study did not collect data on ferritin levels, the limited number of patients with abnormal hemoglobin levels regardless the VWD type, coupled with the absence of clinically significant anemia, supports the assumption that this factor likely did not impact the HRQoL in this study. Nevertheless, we compared the overall scores of patients treated or not treated with iron and observed only a trend toward poorer score in the SF-36 mental domain (MCS) when patients received iron therapy (mainly women), a trend also described in the Canadian cohort among patients with iron deficiency [5].

Finally, the VWD-SAT revealed that patients were satisfied with the care provided in treatment centers and by caregivers whatever their type of VWD. The other dimensions tended to be slightly higher (less satisfied with their treatment) in women and type 3 VWD, especially the treatment burden.

### 4.5 VWD impacts the social health over time

Consistent with the longitudinal stability of bleeding scores, SF-36 scores showed no significant change between M0 and M24. This stability may reflect either a slow physical decline due to the underlying disease or the efficacy of therapeutic management in the French cohort. This finding contrasts with the marked decline in physical role scores observed over 3 years in the Finnish cohort of patients with severe VWD [3]. The VWD-QoL scores demonstrated also longitudinal stability, with no significant changes observed in the overall physical and mental health domains. The only significant deteriorations occurred in relationships with others and with partners, indicating increasing challenges in both social and intimate spheres.

### 4.6 Results are confirmed by the exploratory multivariate analysis

Exploratory multivariate analysis confirmed that disease severity impairs HRQoL. For the VWD-QoL, a negative impact was associated with type 3 VWD and disability status (signs of disease severity). For the SF-36, negative factors were linked to disease severity or its long-term consequences, including joint damage as already described by van Galen et al. [12], longer time since diagnosis (earlier diagnosis in type 3), and prolonged work absence. Multivariate analysis confirmed that being female negatively impacts QoL, a finding consistent with the negative trends observed for women compared with men in both questionnaires.

PRO questionnaires are valuable for assessing HRQoL in VWD clinical studies, although their integration into routine care is limited by time constraints and a lack of automated scoring systems. VWD-specific PROs demonstrate that targeted questions better identify high-impact patient subgroups and delineate specific mental and social limitations. These insights highlight the potential value of complementary interventions—such as physiotherapy, adapted physical activity, and psychosocial support—to improve well-being, sustain social relationships, and facilitate professional participation.

Furthermore, the results underscore the importance of therapeutic patient education for all VWD types (not only type 3), particularly for women, alongside support from patient associations and adapted care pathways. Collectively, these data reinforce the need to strengthen rare disease plans and care networks at both national and European levels.

### 4.7 Study limitations

However, this study has some limitations. Due to the inclusion criteria excluding patients with VWF:Ag levels of ≥ 30 IU/dL for type 1 VWD, the study population represents a group of adults with a high proportion of severe VWD (50% had VWF:RCo level of < 15 IU/dL). Therefore, the results are not generalizable to milder forms of type 1 VWD. As expected, few patients were treated with LTP, and analysis of the impact of initiating LTP during the study could not be evaluated in this study. Since this was not an interventional study, there was a significant dropout rate between inclusion and follow-up. This resulted in fewer HRQoL data collected at M24. However, this observational study reflects current medical practice and treatments. The inclusion of patients with different types ensures a representative sample of the actual clinical situations encountered in France.

## 5 Conclusion

The WiSH-QoL study, particularly its VWD-specific HRQoL assessment, provided valuable insights into the daily lives and well-being of adult patients living with a VWD in France. Indeed, it appears that these QoL studies are linked to the patients’ country of origin, suggesting the significant impact of societal and contextual factors.

It demonstrated the profound impact of VWD across physical, mental, and social health domains, highlighting the need for tailored care—especially for patients with severe disease and women. While implementing full PRO questionnaires in routine practice remains challenging, selecting key items could help clinicians address not only physical symptoms but also mental health and social challenges. Furthermore, the documented impact on employment and social relationships highlights the necessity of advancing VWD care to support these essential dimensions of HRQoL.
