# Supplementary material for: Health-related quality of life in adults with von Willebrand disease: results of the French real-life Willebrand study on health-related quality of life
Source: Res Pract Thromb Haemost. 2025 Dec 30;10(1):103324. doi: 10.1016/j.rpth.2025.103324 (PMC12887095; doi:10.1016/j.rpth.2025.103324)
Supplement: Supplementary Material [file mmc1.docx]

**Supplemental materials**

# **Table S1: SF-36 dimensions**

| **Dimensions*** | **No of items** | **Underlying concept** | **Summary scores** |
| --- | --- | --- | --- |
| Physical functioning | 10 | Limitations in daily activities (e.g. when walking, washing, dressing, shopping, cleaning...) due to health problems | **Physical component summary**  **(PCS)** |
| Role physical | 4 | Impact of physical limitations on work or daily activities |  |
| Bodily pain | 2 | Intensity of pain and its impact on work and domestic activities |  |
| General health | 5 | Individual subjective assessment of general health |  |
| Vitality | 4 | Energy levels or fatigue | **Mental component summary**  **(MCS)** |
| Social functioning | 2 | Limitations on social activities (e.g. meeting friends) due to health problems |  |
| Role emotional | 3 | Impact of emotional problems on work or daily activities |  |
| Mental health | 5 | Psychological distress: presence of depressive or nervous feelings |  |

* *Health transition (1 item): The respondent is asked to rate their current health status compared to their health status one year ago. This question remains separate from the 8 subscales and is not scored.*

# **Table S2: VWD-specific questionnaire (VWD-QoL and VWD-SAT) dimensions**

| Dimensions | N° of items | Underlying concepts |
| --- | --- | --- |
| VWD-QoL scale | |  |
| Treat | 11 | Treatment for VWD (if applicable) |
| Complaint | 9 | Side effects caused by treatment for VWD (if applicable) |
| Physical | 7 | Physical health |
| Feeling | 13 | Feelings about VWD |
| View | 6 | Impact of VWD on self-perception |
| Family | 5 | Relationships with the family in relation to the disease |
| Others | 5 | Relationships with other people in relation to the disease |
| Sport | 5 | Practice of sport and leisure |
| Work | 4 | Work or school life |
| Deal | 7 | Attitude to deal with VWD |
| Hospital | 6 | Relationships with the hospital and hospital staff |
| Future | 4 | View of the future |
| Sex | 3 | Relationship and partnership |
| TOTAL | All VWD-QoL dimensions equally contribute to the total score | |
| VWD-SAT scale | |  |
| Ease | 10 | Simplicity and convenience of treatment use |
| Efficacy | 6 | Treatment efficacy |
| Burden | 4 | The burden of treatment |
| Specialist | 7 | Specialist doctor / nursing staff caring for the patient |
| Centre | 5 | Hospital care |
| General | 2 | General satisfaction with treatment |
| TOTAL | All VWD-SAT dimensions equally contribute to the total score | |

# **Table S3: Missing data rules**

| **Questionnaires** | **Missing data rules** |
| --- | --- |
| **SF-36** | The «Half-Scale Rule» procedure was applied to substitute missing data.    The dimension was calculated if at least half of the dimension’s items was answered (or half +1/2 in case of odd number of items), and missing data were substituted by an estimation based on the answers to the other items.  This approach was not applied to the PF dimension due to the hierarchical nature of the items composing it; instead, the «item response theory» (IRT) procedure is applied. |
| **VWD-QoL &**  **VWD-SAT** | The rules depended on the number of items per domain.  Domains or Total scores were not calculated if one, more than one and more than 2 items were missing in 1 or 2-items, 3-to-9-items domains and 10+items domains respectively. If fewer items were missing, the domain score and the total score were calculated by imputing item’s answer with the mean of the domain’s non-missing answers. |

# **Table S4: Exploratory univariate and multivariate analyses and independent variables used**

For each of the three exploratory models testing the sensitivity of generic and VWD-specific questionnaires, univariate analyses were first carried out, and the variables with a p-value lower than 0.20 were retained for a multivariate regression model. The variables with a p-value lower than 0.05 in the multivariate model after a backward selection of variables were considered statistically significant. Regression coefficients and their associated 95% confidence intervals and p-values are presented. All tests were bilateral, with p-values lower than 0.05 considered statistically significant

| Variables | Categories |
| --- | --- |
| VWD types | Type 1, Type 2, Type 3, Type unknown |
| Sex | Male, Female |
| Age groups | 18-49 years, ≥50 years |
| Marital status | Married, living with a partner, single or divorced/separated, widow(er) |
| Professional status | Student or retired, Extended work stoppage, Full or part-time job, No professional activity, Intermittently employed, Other |
| Disability status | Yes/No |
| Time to diagnosis | months |
| Body Mass Index categories | Underweight (<18), Normal (18-24.9), Overweight (25-29.9), Obese (≥30) |
| Tosetto bleeding score | number |
| Viral infection (HIV and/or HBV and/or HCV) | Yes/No |
| Concomitant pathology (indicator by pathology or number of organs involved or indicator of at least one other pathology) | Yes/No |
| Concomitant psychiatric pathology | Yes/No |
| Gastrointestinal Haemorrhage | Yes/No |
| Joint lesion | Yes/No |

# **Table S5: Concomitant pathologies according to age groups at inclusion**

| **At Inclusion (M0)** | **18-50 yo (N=153)** | **51-64 yo (N=42)** | **≥65 yo (N=29)** | **All patients* (N=224)** |
| --- | --- | --- | --- | --- |
| **At least one concomitant pathology N (%)** | **91 (59.5)** | **36 (85.7)** | **28 (96.6)** | **155 (69.2)** |
| Cardiovascular system | 12 (7.8) | 12 (28.6) | 23 (79.1) | 47 (21.0) |
| Respiratory system | 11 (7.2) | 1 (2.4) | 2 (6.9) | 14 (6.3) |
| Urinary and genital tract | 9 (5.9) | 5 (11.9) | 5 (17.2) | 19 (8.5) |
| Cancer | 0 | 4 (9.52) | 5 (17.24) | 9 (4.02) |
| ENT | 10 (6.5) | 2 (4.8) | 7 (24.1) | 19 (8.5) |
| Skin | 6 (3.9) | 2 (4.8) | 3 (10.3) | 11 (4.9) |
| Psychiatric | 4 (2.6) | 5 (11.9) | 2 (6.9) | 11 (4.9) |
| Skeletons and muscles, excluding complications directly or indirectly related to VWD | 20 (13.1) | 15 (35.7) | 12 (41.4) | 47 (21.0) |
| Digestive and hepatic systems | 25 (16.3) | 10 (23.8) | 7 (24.1) | 42 (18.8) |
| Immune system | 7 (4.6) | 0 | 1 (3.5) | 8 (3.6) |
| Nervous system | 7 (4.6) | 1 (2.4) | 2 (6.9) | 10 (4.5) |
| Metabolic disorders | 17 (11.1) | 8 (19.1) | 11 (37.9) | 36 (16.1) |
| Other | 33 (21.6) | 12 (28.6) | 10 (34.5) | 55 (24.6) |

VWD: Von Willebrand Disease, yo: years old, ENT: Ears, Nose and Throat
*no statistical test was performed between the age groups due to the high granularity of the data

# **Table S6: Socio-demographic characteristics according to VWD types at inclusion**

| **At Inclusion (M0)** | **Type 1**  **(N=44)** | **Type 2**  **(N=126)** | **Type 3**  **(N=14)** | **Type unknown**  **(N=7)** | **All patients**  **(N=224)** |
| --- | --- | --- | --- | --- | --- |
| **Marital status** **N**  Living with a partner, n (%) | **41**  29 (71) | **119**  76 (64) | **13**  7 (54) | **7**  5 (71) | **180**  117 (65) |
| **Place of residence N**  Rural, n (%)  Urban, n (%) | **44**  15 (34)  29 (66) | **124**  39 (31)  85 (69) | **14**  3 (21)  11 (79) | **7**  5 (71)  2 (29) | **189**  62 (33)  127 (67) |
| **Disability status N**  Yes, n (%) | **44**  1 (2) | **119**  10 (8) | **14**  4 (29) | **7**  1 (14) | **184**  16 (9) |
| **Therapeutic education N**  Therapeutic education program done, n (%)  **N**  VWF infusion at home, n (%) | **42**  2 (5)  **28**  6 (21) | **119**  11 (9)  **78**  19 (24) | **14**  8 (57)  **14**  13 (93) | **7**  -  **5**  1 (20) | **182**  21 (12)  **125**  39 (31) |
| **Highest education levels N**  Lower secondary education (ISCED 2), n (%)  High school diploma (ISCED 3), n (%)  Professional diploma (ISCED 5), n (%)  University diploma (ISCED 6 to 8), n (%) | **44**  1 (2)  24 (55)  12 (27)  7 (16) | **122**  10 (8)  52 (43)  21 (17)  39 (32) | **14**  1 (7)  7 (50)  4 (29)  2 (14) | **7**  1 (14)  3 (43)  1 (14)  2 (29) | **187**  13 (7)  86 (46)  38 (20)  50 (27) |
| **Socio-professional category N**  Farmer, n (%)  Craftsman, trader, entrepreneur, n (%) Executive, liberal profession, n (%)  Employee, n (%)  Worker, n (%)  Other, n (%) | **41**  1 (2)  1 (2)  8 (20)  25 (61)  0  6 (15) | **110**  2 (2)  4 (3)  25 (23)  58 (53)  9 (8)  12 (11) | **12**  0  0  3 (25)  7 (58)  0  2 (17) | **6**  0  0  2 (33.3)  4 (66.7)  0  0 | **169**  3 (2)  5 (3)  38 (22)  94 (56)  9 (5)  20 (12) |
| **Working status N**  Extended work stoppage, n (%)  Student or retired, n (%) No professional activity, n (%) Intermittently employed, full or part-time work, n (%)  Other, n (%) | **44**  1 (2)  13 (30)  7 (16)  23 (52)  0 (0) | **124**  1 (1)  31 (25)  17 (14)  72 (58)  3 (2) | **14**  2 (14)  4 (29)  1 (7)  7 (50)  0 (0) | **7**  0 (0)  1 (14)  0  6 (86)  0 (0) | **189**  4 (2)  49 (26)  25 (13)  108 (57)  3 (2) |
| **Absenteeism from work or inability to perform a household task (days) per year N**  mean (SD)  median [Min; Max] | **35**  3.9 (7.4)  0 [0; 30] | **95**  12.4 (52.9)  0 [0; 365] | **12**  61.9 (77.5)  28 [0; 240] | **6**  21.7 (48.3)  0 [0; 120] | **148**  14.8 (50.5)  0 [0; 365] |
| SD: Standard deviation, Max: Maximum; Min: Minimum. VWF: von Willebrand Factor ISCED: [International Standard Classification of Education](https://ec.europa.eu/eurostat/statistics-explained/index.php/International_Standard_Classification_of_Education_(ISCED)) | | | | | |

# **Table S7: Medical events during the 24 months follow-up period**

| **24 months follow-up period** | **Type 1  (N=49)**  **ne n (%)** | | **Type 2  (N=152)**  **ne n (%)** | | **Type 3  (N=15)**  **ne n (%)** | | **Type UNK**  **(N=8)**  **ne n (%)** | | **All patients  (N=224)**  **ne n (%)** | |
| --- | --- | --- | --- | --- | --- | --- | --- | --- | --- | --- |
| **All medical events** | 62 | 21 (43) | 263 | 95 (63) | 90 | 15 (100) | 10 | 3(38) | 425 | 134 (60) |
| **Number of events/ patient**  median [range] | 1 [1-24] | | 2 [1-10] | | 6 [1-21] | | 2 [1-7] | | 2 [1-24] | |
| **Number of patients with at least 1 bleeding** n (%) | 6 (12) | | 68 (45) | | 14 (93) | | 2 (25) | | 90 (40) | |
| **Number with at least 1 VWF infusion** n (%) [Number on LTP] | 14 (29)  [2] | | 62 (41)  [2] | | 15 (100)  [9] | | 3 (38)  [0] | | 94 (42)  [13] | |
| **Minor bleeding**  **(% on all events)** | 30  (48) | 6 | 114  (43) | 64 | 60  (66) | 13 | 6  (60) | 1 | 210  (49) | 84 (38) |
| *VWF*  *DDAVP*  *Other*† | *1*  *2*  *0* | *1*  *1*  *0* | *54*  *8*  *14* | *20*  *6*  *8* | *58*  *0*  *19* | *12*  *0*  *4* | *0*  *0*  *0* | *0*  *0*  *0* | *113*  *10*  *33* | *33*  *7*  *12* |
| **Major bleeding**^¶^  **(% on all events)** | 0  (0) | 0 | 17  (6) | 9 | 7  (8) | 5 | 1  (10) | 1 | 25  (6) | 15 (7) |
| *VWF*  *Other*† | *0*  *0* | *0*  *0* | *15*  *11* | *8*  *6* | *7*  *4* | *5*  *3* | *1*  *0* | *1*  *0* | *23*  *15* | *14*  *9* |
| **Surgery***  **(% on all events)** | 22  (35) | 17 | 67  (25) | 47 | 10  (11) | 6 | 3  (30) | 3 | 102  (24) | 73 (33) |
| *VWF*  *Other*† | *15*  *1* | *12*  *1* | *55*  *8* | *40*  *7* | *10*  *1* | *6*  *1* | *3*  *0* | *3*  *0* | *83*  *10* | *61*  *9* |
| **Invasive procedure**  **(% on all events)** | 10  (16) | 6 | 65  (25) | 35 | 13  (14) | 5 | 0 | 0 | 88  (21) | 46 (21) |
| *VWF*  *Other*† | *2*  *0* | *2*  *0* | *43*  *7* | *22*  *7* | *11*  *1* | *5*  *1* | *0*  *0* | *0*  *0* | *56*  *8* | *29*  *8* |

ne: number of medical events; n: number of patients LTP: Long-Term Prophylaxis, DDAVP

†other treatment including labile blood products, platelet concentrates, antifibrinolytic, tranexamic acid, nonsteroidal anti-inflammatory drugs or corticosteroids; VWF: von Willebrand Factor

^¶^ Major bleeding was described as having required the patient to remain hospitalized at least 3 days

* Surgery including delivery

# **Table S8: SF-36 and VWD-QoL multivariate analysis results (N=169/186)**

| Dependent variable | Parameter | β Coefficient | 95% CI | p-value |
| --- | --- | --- | --- | --- |
| SF-36  Physical Component score | *Intercept* | *54.8* | *[52.6; 57.1]* | *<0.001* |
|  | **Time since diagnosis**  *(in years)* | -0.1 | [-0.2; -0.0] | 0.037 |
|  | **Joint lesion**  *Yes (vs No)* | -11.8 | [-17.1; -6.5] | <0.001  <0.001 |
|  | **Professional status**  Extended work stoppage (vs full or part-time job)  No professional activity  *Intermittently employed*  *Student or retired*  *Other* | -11.0  -3.8  *-2.3*  *0.4*  *-7.3* | [-19.5; -2.4]  [-7.4; -0.1]  *[-13.9; 9.2]*  *[-2.6; 3.3]*  *[-16.9; 2.2]* | 0.033  0.012  0.044  *0.690*  *0.795*  *0.132* |
| VWD-QoL  Total score | *Intercept* | *23.3* | *[19.0; 27.6]* | *<0.001* |
|  | **Phenotype**  Type 3 (vs Type 1)  *Type 2 (vs Type 1)*  *Type unk (vs Type 1)* | 13.9  *-2.5*  *-1.6* | [5.8; 22.0]  *[-7.5; 2.5]*  *[-11.3; 8.0]* | <0.001  *<0.001*  *0.320*  *0.74* |
|  | **Disability status**  *Yes (vs No)* | 8.3 | [1.8; 14.8] | 0.013  0.013 |
|  | **Gender**  *Male (vs Female)* | -5.3 | [-9.7; -0.8] | 0.021  0.021 |

CI: Confidence Interval, unk: unknown
